# Methods to Analyse Time-to-Event Data: The Kaplan-Meier Survival Curve

**DOI:** 10.1155/2021/2290120

**Published:** 2021-09-20

**Authors:** Graziella D'Arrigo, Daniela Leonardis, Samar Abd ElHafeez, Maria Fusaro, Giovanni Tripepi, Stefanos Roumeliotis

**Affiliations:** ^1^Institute of Clinical Physiology (IFC-CNR), Clinical Epidemiology and Physiopathology of Renal Diseases and Hypertension of Reggio Calabria, Italy; ^2^Epidemiology Department, High Institute of Public Health-Alexandria University, Alexandria, Egypt; ^3^National Research Council (CNR)–Institute of Clinical Physiology (IFC), Pisa, Italy; ^4^Department of Medicine, University of Padova, Italy; ^5^Division of Nephrology and Hypertension, 1st Department of Internal Medicine, AHEPA Hospital, School of Medicine, Aristotle University of Thessaloniki, Thessaloniki, Greece

## Abstract

Studies performed in the field of oxidative medicine and cellular longevity frequently focus on the association between biomarkers of cellular and molecular mechanisms of oxidative stress as well as of aging, immune function, and vascular biology with specific time to event data, such as mortality and organ failure. Indeed, time-to-event analysis is one of the most important methodologies used in clinical and epidemiological research to address etiological and prognostic hypotheses. Survival data require adequate methods of analyses. Among these, the Kaplan-Meier analysis is the most used one in both observational and interventional studies. In this paper, we describe the mathematical background of this technique and the concept of censoring (right censoring, interval censoring, and left censoring) and report some examples demonstrating how to construct a Kaplan-Meier survival curve and how to apply this method to provide an answer to specific research questions.

## 1. Introduction

The clinical research in the areas of oxidative stress and biological consequences of aging and alterations of immune function and metabolism commonly demands the application of specific statistical techniques aimed at investigating the strength of the relationship between certain risk factors (for example, oxidized low density lipoprotein) and adverse outcomes (for example, death and cardiovascular events). The analysis of time-to-event data is of paramount importance in clinical and epidemiological research [1]. In the setting of prospective or retrospective cohort studies [2], survival data refer to the time spanning from a well-defined date (which coincides with the date of enrolment or starting the observation of an individual) to the occurrence of a given clinical endpoint (for example, death, cardiovascular events, and relapse of a given disease).

Thus, survival times do not always correspond to the actual survival of a given individual with mortality being the event of interest. Survival analysis is aimed at assessing the trend of a given event occurrence as a function of time (i.e., a single survival curve plotted against time in days, months, or years) and comparing survival curves between two (for example, between treated and untreated patients or exposed/unexposed individuals to a given risk factor) or more than two groups of individuals. Survival data require peculiar methods of analysis because not all patients enrolled in a given study experience the event of interest. For example, in a cohort study with a cardiovascular death as the primary endpoint [3], someone may drop out due to change residence, death due to noncardiovascular causes (for example, cancer), or he might complete the scheduled time period without experiencing the event of interest. These incomplete observations are called “censored observations” which can occur during or at the end of the study. Three types of censoring exist. The first is right censoring which is the most common one and occurs when a patient is followed up over a time period without having the event of interest. Thus, the survival time is incomplete at the right side of the follow-up period. For this patient, we know that the event of interest does not occur till the censoring date but we do not know whether the event will occur thereafter or not. The second is interval censoring which describes the situation when the event of interest happens within an interval between two dates but we do not know exactly the date. The third is left censoring that happens when an individual belonging to a given cohort is known to have the event before a specific date, but the time period between the occurrence of the event and the specific date remains unknown.

In 1958, Edward L. Kaplan and Paul Meier wrote an article describing how to deal with censored observations, thus posing the basis of the Kaplan-Meier (K-M) survival analysis. The Kaplan-Meier survival method allows to analyse the time to the first event, and one of the peculiarities of this technique is that the time intervals are dictated by the occurrence of the event of interest. For example, a hypothetical cohort study with the occurrence of myocardial infarction over 1-year time period is the primary endpoint; if a patient experiences two myocardial infarctions (the first after 6 months and the second one after 10 months from the enrolment), the Kaplan-Meier analysis will only take into consideration the first occurrence of the event (i.e., the myocardial infarction at 6 months).

Three fundamental assumptions should be carefully considered when constructing a K-M survival curve. The first assumption is that censoring should be nonuninformative, that is, unrelated to the study outcome. This means that censored observations should have the same probability of the event (after censoring) as those remaining under observation. This implies that baseline prognostic characteristics of patients who are censored should be similar to those of patients who remain under observation. The second assumption is that the survival probabilities should be the same for individuals recruited early and late in a given study. The third assumption is that day, month, and year of the occurrence of a specific event of interest must be available to provide accurate survival estimates. Another characteristic of the Kaplan-Meier method is that it does not allow to adjust for confounding; a problem of particular relevance in observational studies is aimed at assessing causal relationships [4].

In this paper, we provide a series of examples useful to understand and interpret a K-M survival curve.

## 2. Mathematical Background of the Kaplan-Meier Analysis

The minimal set of information to construct a Kaplan-Meier survival curve includes the time to the event of interest (for example, days, months, and years) and the binary variable indicating patients' status (presence/absence of the condition) at that point in time. The time between the enrolment and the terminal event/end of observation is represented by a random variable *T* (*T* > 0) defined as “survival time.” By considering *n* patients and *t*_1_, *t*_2_, ⋯., *t*_*j*_ (*j* ≤ *n*) the observed times to event, the survival time, at time *i*, is *T*_*i*_ = *t*_*j*_ − *t*_*i*_. Basically, the Kaplan-Meier method estimates the conditional probability of survival calculated at specific time points dictated by the occurrence of the event. The conditional probability (or cumulative probability or cumulative survival) is the probability $\hat{\left(S\left(t\right)\right)}$ that a patient survives *x* days after entering a study conditional to the fact that the same patient survives the days before. For example, in a hypothetical setting in which a patient admitted to an intensive care unit survives for three days, the cumulative survival (calculated by the product rule of conditional probabilities) is the product of survival probabilities at day 1 (*p*_1_), at day 2 (*p*_2_), and at day 3 (*p*_3_), that is:

(1) $$\begin{array}{c} \hat{S\left(t\right)}=p_{1}*p_{2}*p_{3}. \end{array}$$

If we indicate with *d*_*j*_, the number of subjects present the event of interest (for example, death) at time *t*_*j*_ and with *n*_*j*_, the number of individuals at risk at time *t*_*j*_. The individual probability $\hat{\left(q\right)}$ to die at *t*_*j*_ conditional to be alive at *t*_*j*−1_ is:

(2) $$\begin{array}{c} \hat{q}=\frac{d_{j}}{n_{j}}. \end{array}$$

Therefore, the probability of surviving at time *t*_*j*_ is as follows:

(3) $$\begin{array}{c} \hat{p_{j}}=1-\hat{q_{j}}=\frac{n_{j-}d_{j}}{n_{j}}. \end{array}$$

By multiplying the estimates of the conditional probabilities of surviving, we obtain the estimate of the cumulative probability of living beyond the instant *t*_*j*_:

(4) $$\begin{array}{c} \hat{S\left(t\right)}=\underset{j/t_{j\leq t}}{\prod }\hat{P_{j}}. \end{array}$$

This latter formula represents the Kaplan-Meier estimator with asymptotic variance estimated by Greenwood formula by:

(5) $$\begin{array}{c} \text{Var}\left[\hat{S\left(t\right)}\right]=S{\left(t\right)}^{2}\underset{\frac{j}{t_{j\leq t}}}{\sum }\frac{d_{j}}{n_{j}\left(n_{j}-d_{j}\right)}, \end{array}$$

that is inversely proportional to the number of subjects at risk.

The Kaplan-Meier estimator of survival probability is represented by a curve, which starts from 1 and decreases over time. The magnitude of the steps depends on the number of events and the number of subjects at risk. To compare the survival distributions of two or more groups of subjects who undergo to different treatments or exposure, the log-rank test (a nonparametric, rank-based test) is used. We consider a series of patients randomized 1 : 1 to an experimental treatment and to a control treatment. The study end-point is death. Under the null hypothesis (H_0_) that the two treatments have the same efficacy, the number of patients who died is expected to be approximately the same in both groups. Vice-versa, the alternative hypothesis (H_1_) is that the death rate differs between the two study arms, implying that a difference between expected and observed deaths exists. Thus, the log rank test compares the observed numbers of deaths in each group to the death rate expected if the null hypothesis was true. The log rank statistic is approximately distributed as a chi-square test statistic with degree of freedom corresponding to the number of comparison groups-1:

(6) $$\begin{array}{c} \chi^{2}=\sum \frac{{\left(\sum O_{jt}-\sum E_{jt}\right)}^{2}}{\sum E_{jt}}, \end{array}$$

where *ΣO*_*jt*_ is the sum of the observed number of events in the *j*^th^ group over time (*j* = 1, 2) and *ΣE*_*jt*_ is the sum of the expected number of events in the *j*^th^ group over time.

To calculate the expected numbers of events, we estimate the proportion of events occurring at each time (*O*_*t*_/*N*_*t*_) using data from both groups combined under the assumption of no difference in survival (i.e., assuming the null hypothesis is true). We multiply these estimates by the number of participants at risk at that time in each of the comparison groups (*N*_1*t*_ and *N*_2*t*_ for groups 1 and 2, respectively). For example, we consider 10 patients at risk in group 1 and 10 patients in group 2 and 1 the total number of expected events at time *t* (for example, 14 months) is *E*_1*t*_ = *N*_1*t*_∗(*O*_*t*_/*N*_*t*_) = 10∗(1/20) = 0.500 and *E*_2*t*_ = *N*_2*t*_∗(*O*_*t*_/*N*_*t*_) = 10∗(1/20) = 0.500, respectively. By using this information and the formula reported above, a *χ*^2^ can be calculated, and a *p* value for this test can be derived by an opposite table according to the value of *χ*^2^ and the degrees of freedom.

### 2.1. Example 1

**Figure 1:**
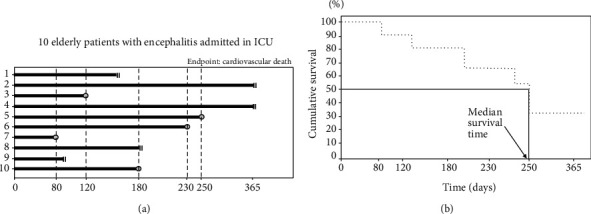
Hypothetical example useful to understand how to build up a Kaplan-Meier curve (see example 1).

In a hypothetical study, ten elderly patients with encephalitis (an inflammatory condition of the brain) admitted to intensive care unit (ICU) were followed up over a 365-day period. The primary endpoint was cardiovascular death. During the follow-up period, 5 patients died. The aim of this study is to build up the survival curve of the incidence of cardiovascular death in the study population. The K-M survival curve plots the cumulative probability of survival in a given period as a function of time. Before constructing the K-M curve, a fundamental prerequisite is to know the exact time of the event occurrence, the number of subjects at risk, the number of events in the period, and the censored observations (e.g., individuals who are lost to the follow-up or patients who leave the study for events other than that of interest). Patients who are censored remain in the analysis until information about their status are available. For each time interval, the survival probability in each period is calculated as the number of subjects who survive divided by the number of patients at risk at the beginning of the period. Therefore, according to the product rule of probabilities, the cumulative survival after each period (except the first one) is calculated by multiplying the probabilities of survival of the previous periods. We consider in detail data of our hypothetical example. Patients 1, 8, and 9 were censored: the first two were lost to the follow-up and the last one died from another cause (cancer). Patients 2 and 4 were alive till the end of the observation. Five patient died of CV causes at different time: patient 3 died at 120 day, 5 at 250 days, 6 at 230 days, 7 at 80 days, and 10 at 180 days. In the graph, the double vertical lines indicate the censored observations, whereas the grey circles indicate patients with the event of interest (Figure 1(a)). To build up the K-M curve, we divide time into intervals corresponding with the occurrence of each event.  Table 1 reports the information we need to construct the K-M curve.

In the 1^st^ interval, there is only one patient who died of CV causes; in the 2^nd^ one, there are 1 patient with the event of interest and 1 patient censored, and so on. To calculate the survival probability, we consider the numerator as the difference between the number of patients at risk and the number of patients who died and at the denominator the number of patients at risk at the beginning of the period. For the first two periods, we have:

(7) $$\begin{array}{c} S\text{urvival probability in the }1^{\text{st}}\text{ period}=\left[\frac{\left(10-1\right)}{10}\right]=0.900\;\left(90\%\right),\\ \text{Survival probability in the }2^{\text{nd}}\text{ period}=\left[\frac{\left(9-1\right)}{9}\right]=0.890\left(89\%\right). \end{array}$$

Finally, we calculate the cumulative survival across the whole study period as the product of survival probabilities of each period. In the first interval, both survival probability and cumulative probability coincide. At the end of the second period, the cumulative survival was calculated as 0.900∗0.890 = 0.801 (80.1%). At the end of the third period, the cumulative survival was calculated as 0.900∗0.890∗0.857 = 0.687 (68.7%). To build up the K-M survival graph, we report in the abscissa axis the follow-up time (specifying if the units of measurement are days, month, or years) and in the ordinate axis the cumulative survival (i.e., probabilities with values ranging from 0 to 1) (Figure 1(b)). The dimension of horizontal lines (i.e., those parallel to the abscissa axis, Figure 1(b)) corresponds to the duration of the interval between consecutive events, whereas the vertical distances represent the change in the cumulative survival. By using a graphical approach, we can calculate the median survival time, i.e., the time which corresponds to a cumulative survival of 50% (Figure 1(b)). In our case, the median survival time is 250 days. We can construct a single survival curve, two, or more than two survival curves. For example, we can compare the survival of two groups of patients (exposed/not exposed to a given risk factor such as with/without diabetes, males/females, and smokers/nonsmokers,). The test used to compare two or more curves is the log rank test. A detailed description of this test is reported elsewhere [5].

### 2.2. Example 2

**Figure 2:**
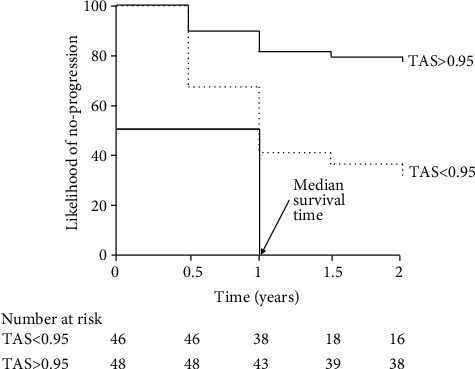
Kaplan-Meier survival curves on the effects of total antioxidant status (TAS) on visual field progression (see example 2). Redrawn from Ref. 6.

To illustrate the application of the K-M method, we consider a prospective cohort study by Li et al. [6]. In this paper, the authors investigated the relationship between oxidative stress biomarkers and visual field progression in patients with primary angle closure glaucoma (PACG). Ninety-four patients with PACG were followed up for at least two years, with periodic visits every 6 months. In these patients, the levels of total antioxidant status (TAS—an oxidative stress biomarker) have been measured. Forty-three patients (45.7%) had progression of glaucoma as assessed by the visual field. Here, we focus on the survival analysis of the relationship between TAS and visual field progression. The authors categorized patients into two groups according to the median value of TAS at baseline (below/above 0.95). As shown in Figure 2, patients with low TAS (<0.95) had a significantly higher percentage of PACG progression (log-rank test *p* < 0.0001) than those whit TAS > 95. Of note, the two curves diverge from 6 months onwards. In patients with TAS < 0.95, the median survival time is 12 months. The cumulative survival does not cross 50% in patients with TAS > 0.95; therefore, the median survival time is not calculable. Below the graph, the number of patients at risk at relevant points in time are also reported. This information must be reported because it represents the fundamental information to interpret a survival curve. The results of this study generate the hypothesis that TAS is a useful biomarker to stratify the risk of progression in patients with PACG.

### 2.3. Example 3

**Figure 3:**
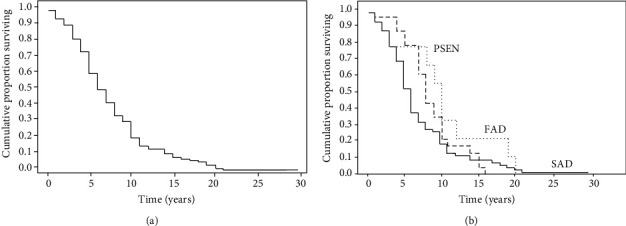
Kaplan-Meier survival curves of all 103 Alzheimer's disease (AD) patients and comparison between curves (see example 3). Redrawn from Ref. 7.

We consider another example in which the authors studied the overall pattern of survival [7]. In the setting of a retrospective study, the authors considered 103 patients with Alzheimer's disease (AD), i.e., the fraction of patients who survive for a certain period after onset of dementia. Mean disease duration of the 103 AD cases was 7.1 years. Information on familial Alzheimer's disease (FAD), on sporadic Alzheimer's disease (SAD), and on presenilin (PSEN) genes were also collected to investigate the effect of FAD, SAD, and PSEN on death rate in these patients by the analysis of the Kaplan-Meir survival curves. Twenty-five percent of cases died within four years, 50% within 6.9 years, and 75% within 10 years after onset of Alzheimer's disease. In the whole group of patients (*n* = 103), the cumulative survival was about 60% at 5 years, 20% at 10 years, and about 10% at 15 years (Figure 3(a)). First of all, the authors compared the survival curves of FAD and SAD patients by the Gehan-Wilcoxon (G-W) two-sample test [8], and a significant difference in mortality rate between the two groups was found (G − W = 2.51, *p* < 0.05). When considering more than two groups, the mortality rate among groups was compared using the log rank test. The survival graphs (Figure 3(b)) showed that patients with PSEN genes survived longer than those with FAD and SAD, and the difference among the three survival curves was highly significant (log rank test = 7.13, *p* < 0.01). The effect of PSEN genes on mortality is of interest because of the role of presenilin in mitochondrial oxidative stress and neurodegeneration [9].

As described above, in addition to the log rank test, the Gehan-Breslow-Wilcoxon can be used to test the statistical significance between the Kaplan-Meir curves. The Gehan-Breslow-Wilcoxon method gives more weight to deaths at early time points. However, it is important noting that the results of this test can be misleading when a large fraction of patients are censored early on. In contrast, the log-rank test gives equal weight to all time points [5].

### 2.4. Example 4

Multiple myeloma (MM) is a type of plasma cell neoplasm. In this condition, the overproduction of intracellular reactive oxygen species (ROS) accompanies malignant transformation to oncogene activation and/or enhanced metabolism in tumor cells. As a consequence, these cells possess higher levels of ROS and lower levels of antioxidant molecules compared to their normal counterparts. Unbalanced production of ROS leads to oxidative stress which could exert a toxic effect for the cell [10]. There is scientific evidence that antioxidant defense confers resistance to high-dose melphalan in MM cells, supporting that redox status in multiple myeloma cells could be determinant for patients' response to melphalan [11].

**Figure 4:**
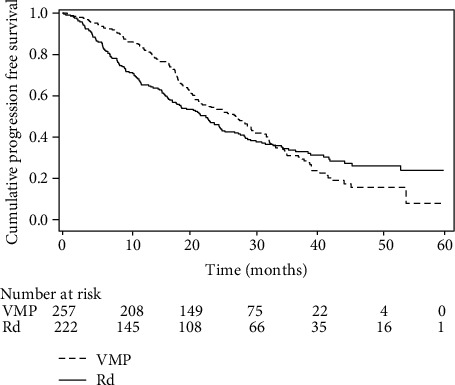
Kaplan-Meier survival curves for progression-free survival in VMP- and Rd-treated patients (see example 4). Redrawn from Ref. 12.

Here, we consider a paper [12] in which the authors compared patients' data (*n* = 479) from two randomized phase III trials to assess the impact of melphalanprednisone plus bortezomib (VMP; *N* = 257) vs. lenalidomide and low-dose dexamethasone (Rd; *N* = 222) on progression-free survival (PFS) in elderly newly diagnosed multiple myeloma patients. Three hundred and six patients had disease progression or died during the follow-up period (median 32 months). By looking at the Kaplan-Meier survival curve, it is possible to note that while VMP significantly reduced the disease progression rate between enrolment and 12 months of follow-up, no difference between the two schedules was found between 12 and 32 months. After 32 months, Rd-treated patients had a lower incidence of disease progression (Figure 4). The authors conclude that time plays a crucial role in interpreting the effect of MPV with respect to Rd on the PFS in elderly newly diagnosed multiple myeloma patients.

### 2.5. Example 5

**Figure 5:**
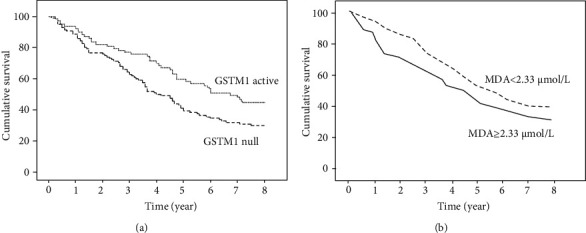
Kaplan-Meier survival curves for overall survival patients with ESRD (see example 5). Redrawn from Ref. 13.

Suvakov et al. [13] investigated the polymorphisms of the gene GST (glutathione S-transferase) as predictors of survival in a series of patients with end-stage renal diseases (ESRD). On the basis of the consolidated notion that glutathione S-transferases (GST) is a well-established antioxidant, the authors analysed a surrogate of this biomarker, the polymorphisms of the GST gene, in order to assess the prognostic role of this SNP for survival in patients with ESRD. They also measured other oxidative biomarkers such as malondialdehyde (MDA). These oxidative biomarkers were measured in 199 haemodialysis patients followed up for 8 years. For the purpose of this paper, we focus on the relationship between overall survival and the two oxidative biomarkers mentioned above (Figure 5). Overall, 120 patients died, and of these 62 of cardiovascular causes (51.7%). GSTM1 genotype was categorized in null and active whereas MDA levels as below/above the corresponding median level (2.33 *μ*mol/L). A Kaplan-Meier survival analysis demonstrated that patients with the GSTM1-null genotype had a shorter overall survival (log-rank 5.748, *p* = 0.017; Figure 5(a)) as compared to remaining patients. Accordingly, patients with higher MDA concentrations had a trend towards poorer overall survival in comparison to those with MDA relatively lower (Breslow: 3.766, *p* = 0.052; Figure 5(b)). The authors conclude that these two biomarkers can be useful for risk stratification in in ESRD patients.

## 3. Conclusions

Survival analysis belongs to the family of statistical methods that analyse the distribution of the time of occurrence of a given condition in a certain period of time. Thus, it investigates the incidence of a given event. The term “survival” does not only refer to mortality, i.e., death, but also to any event of interest (e.g., decrease in blood sugar, hospitalizations, and cancer recurrence). Survival studies are carried out by using cohorts, i.e., patients followed-up over time to collect relevant clinical events when they occur and to link such occurrences to a given exposure. To perform a survival analysis, it is necessary to calculate the “survival time,” determined by the difference between the date in which the event occurs or not and the baseline date. Thus, it is essential to know if subjects experience the event of interest or if they are censored. The knowledge of this information is fundamental in order to allow the statistical software to calculate the cumulative probability of the event. Therefore, the Kaplan-Meier method is essentially a way to construct the survival curves as a function of time, thus allowing an immediate perception of the clinical phenomenon being investigated. A Kaplan-Meir curve reports in the ordinate axis the cumulative survival and in the abscissa axis the time period. When constructing a Kaplan-Meir curve, the time intervals are not established in advance but are dictated by the occurrence of each event that determines the duration of the same intervals. An important advantage of the Kaplan-Meier approach is that the method takes into account censoring which occurs when a patient is lost to follow-up for any reason (withdrawal from the study, change of residence, dead for causes other than the event of interest, etc.). If we have more survival curves (stratified by any factor), it is possible to compare them by various methods such as the log-rank test and the Gehan-Wilcoxon (G-W) two-sample test. The Kaplan-Meir analysis does not allow to adjust for confounders. For this reason, while it is well suited to be used in randomized clinical trials [1], it should be considered only as a first level analysis in observational studies aimed at testing causal-effect relationships.

### 3.1. Suggested Software

There are several statistical software that allow to perform time to event analysis by the Kaplan-Meir method. The best known are:

SPSS 23.0 (https://www.ibm.com/support/knowledgecenter/SSLVMB_23.0.0/spss/advanced/idh_kmei.html)

Stata 16 (https://www.stata.com/support/faqs/graphics/gph/graphdocs/kaplan-meier-survival-function/index.html)

SAS (https://support.sas.com/documentation/onlinedoc/stat/142/kaplan.pdf)

MEDCALC (https://www.medcalc.org/manual/kaplan-meier.php)

## Figures and Tables

**Table 1:** Description of the procedure used to calculate the cumulative survival and construct the curve in Figure 1(b).

Interval	Days	Patients at risk	CV deaths (0=no; 1=yes)	Censored	Survival probability	Cumulative survival
1	0-80	10	1	0	0.900	0.900
2	81-120	9	1	1	0.890	0.801
3	121-180	7	1	1	0.857	0.687
4	180-230	5	1	1	0.800	0.549
5	231-250	3	1	0	0.667	0.366
6	251-365	2	0	2	1.000	0.366
